# Supplementary material for: A physically cryptographic warhead verification system using neutron induced nuclear resonances
Source: Nat Commun. 2019 Sep 30;10:4433. doi: 10.1038/s41467-019-12386-0 (PMC6769018; doi:10.1038/s41467-019-12386-0)
Supplement: Supplementary file 1 — SI [file 41467_2019_12386_MOESM1_ESM.pdf]

**Supplementary Note 1**

**Uncertainty range in the inferred pit mass.** Here we consider the scenario where an inspector uses the transmission data from a neutron beam to infer the range of possible pit masses. To frame the problem, let's assume a hollow pit of Weapons Grade Plutonium (WGPu) in a $\delta$-phase of density $\rho \approx 15$g/cm$^3$. We use public domain information about a Soviet thermonuclear warhead, as reported by Fetter et al.[1], which describe a hollow sphere of estimated inner radius of 6.27 cm and an outer radius of 6.7 cm. Using a neutron beam of a radius of 2.5 cm (similar to the settings used in the experiments in this study), the hosts use a WGPu cylindrical encrypting filter of 2.5 cm radius and the length of 6 cm along the beam axis.

Both the pit and the encrypting filter have to be presented for measurements in boxes made of materials that are optically opaque but transparent to neutrons, such as aluminum. The box size could be about 20 cm. Both the pit and the reciprocal are aligned with external fiducial markers on the box exterior, thus allowing for those same markers to be used in beam-target alignments – for more detail see Hecla et al.[2], Supplementary Note 3.

We now assume that the inspector has analyzed the data, has determined the attenuation $A$ and has used $\ln A = -\mu d$, where $\mu$ is the linear attenuation coefficient, to infer the total thickness $d$ of the plutonium. The inspector has then inferred, correctly, that exactly 6.86 cm of WGPu are present along the z-axis of the beam. What are then the possible values of the pit mass that the inspector can infer from these numbers? There are two limiting scenarios that define the lower and upper bounds on the estimates:

**Scenario 0** At one extreme, the null scenario assumes that all the mass is found in the encrypting filter. Thus for this limit the lower bound on the pit mass is $M_{\text{null}} = 0$.

**Scenario 1** Here the inspector assumes the opposite extreme - that all the WGPu mass is in the pit and that the encrypting filter is empty. In this scenario the pit is a hollow sphere, with an outer diameter matching that of the box, i.e. 20cm. In this case the mass of the pit is inferred to be $M_1 = 15 \cdot (4\pi/3)[10^3 - (10 - 6.83/2)^3]$ grams, i.e. $M_1 \approx$45 kg.

Thus the inspector can only infer that the range of the pit mass is in the range of [0,45] kg. This constitutes useless information, as the critical mass of a bare plutonium sphere is $\sim$10 kg[3]. In other words the inspector has inferred numbers that range from the trivial (zero) to absurdly trivial (super-critical). In the discussion above we made an assumption that the transmitted signal is only dependent on the total areal density of plutonium traversed by the beam. In Supplementary Note 3 we use experimental data and Monte Carlo simulations to prove this, showing that various geometric and isotopic pit-filter combinations produce the same signal, as long as the average enrichment and the total combined areal density is kept the same.

In this process the host used an encrypting filter of cylindrical shape of a mass of approximately 1.8 kg. An object of this mass is fully sub-critical, and should not be of any safety concern. In a realistic verification campaign the host will need to produce filters of many thicknesses, furthermore using different thicknesses for different rotations. However this does not mean that the host needs to produce a number of monolithic filters equal to the number of all weapons undergoing dismantlement times the number of random rotations. The filter may consist of thin disks

of plutonium grouped together, allowing the host to modify the thickness by changing the number of the disks. For example, the host can produce 59 disks of 1 mm thickness, 99 disks of 10 $\mu$m thickness, and 10 foils of 1 $\mu$m thickness, allowing a range of thicknesses in the range of [0,6] cm, with a discretization of just 1 $\mu$m. As the protocol cannot resolve thicknesses of $\ll$0.1 mm, this discretization amounts to a continuum, allowing for continuously random filter thickness selections. This single set of disks can be reused between measurements, albeit with different number of disks making up the filter, and will have a total weight of just 1.8 kg of WGPu.

It is necessary to point out that if a 6 cm filter is used, then the inspectors may be able to infer that the pit is not a solid sphere. The diameter of a solid sphere of 3.4 kg, the mass of the example used in this study, is closer to 7.6 cm. To avoid such outcome the host simply needs to increase the thickness of the filter from 6 cm to 6.8 cm. This would require approximately 10% longer measurement time to achieve the equivalent statistics.

As a final consideration it is also important to determine the expected attenuation of the neutron flux by the combined WGPu thickness of 6.86 cm. Using JEFF-3.2 cross section data it is possible to calculate the energy dependent transmission by this filter. Supplementary Figure 1 plots this quantity, showing an average transmission of the order of $\sim$0.2. For comparison, the targets used in the experiments described by this study involved transmissions significantly lower than this number. It is then possible to conclude that a target of this combined thickness will still allow for collection of the necessary statistics in reasonable times, e.g. $\sim$5 minutes. In Supplementary Note 3 we take this analysis further, using experimental data and Geant4 simulations for a WGPu pit

geometry to show that 5 minute long measurements are sufficient to detect a hoax.

## Supplementary Note 2

**Effects of decay heat on Doppler broadening of the resonances** Most of the isotopes in the plutonium pit of a warhead undergo alpha decay, which leads to constant generation of thermal energy. This thermal energy results in a steady state heating of the pit. Different levels of heating in the pit and the encrypting filter may give rise to a difference in resonant line widths due to different extents of Doppler broadening. This could, theoretically, give rise to opportunities of inference of pit material quantities via careful fitting of the absorption lines by the appropriate functional forms. It is possible to determine the extent of this heating, the steady state temperature of the pit, the extent of Doppler broadening, and thus describe the extent of this possible information vulnerability.

The WGPu contains about 94% of $^{239}$Pu, as reported by Mark et al.[3] in Table 1. The nucleus undergoes alpha decay with the half life of 24110 years, emitting an alpha of 5.2 MeV. One can then determine the total power of the heating, from

$$P = E \cdot 0.00012 \cdot \frac{f M N_{\text{Av}}}{A} \ln(2)/\tau_{1/2}$$

where $E$ is the energy of the alpha, $f$ is the enrichment of a particular isotope, $M$ is the mass of the pit (3.4 kg for the example in Supplementary Note 1), $\tau$ is the half life of the isotope. For $^{239}$Pu we find $P_{239} = 6$ Watt. Adding decay from $^{238}$Pu and $^{240}$Pu the total power is $P_\alpha \approx 7$ Watt.

**a**

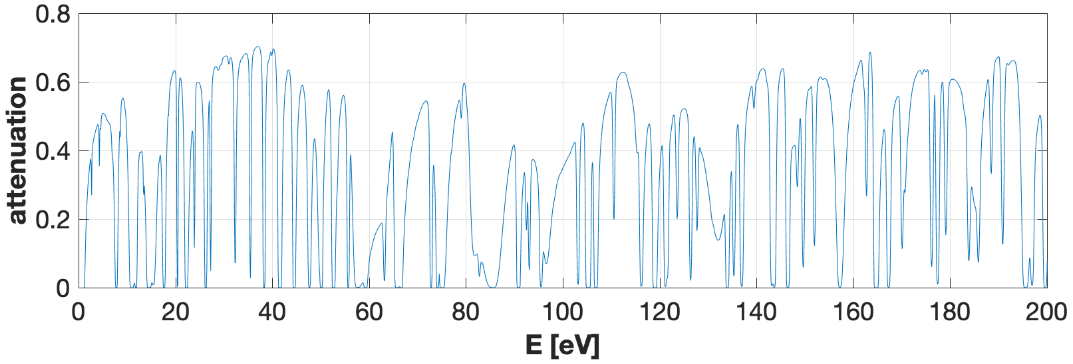


**b**

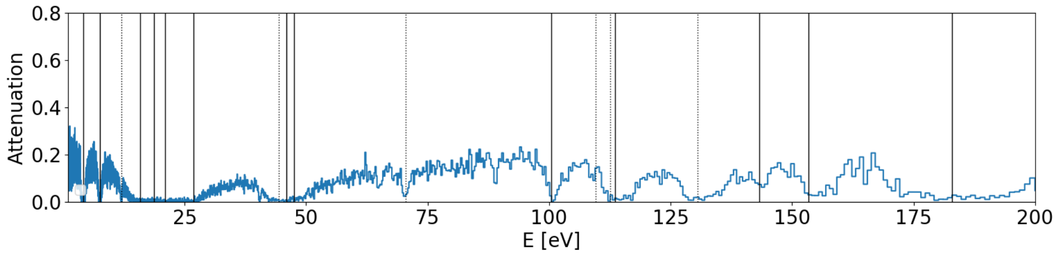


Supplementary Figure 1: Dependence of transmission on the neutron energy. Calculated for (**a**) 6.86 cm of WGPu via analytical calculations based on JEFF-3.2 cross section data, and (**b**) for one of the genuine object samples in this study via experimental data. By comparison to this study the realistic WGPu measurement scenarios will involve larger statistics and thus will involve shorter measurement times.

Approximating the pit as a homogeneous sphere of a constant volumetric heat production, we can now determine the steady state temperature at the surface of the pit, which is determined by the following equation[4]:

$$T_0 - T_\infty = qR/3h$$

where $T_\infty$ is the room temperature, $q$ is the volumetric heat production, $R$ is the outer radius of the pit, and $h$ is heat transfer coefficient, which is approximately 20 W/m$^2$/K for air flowing at 1 m/s, a speed that can be maintained with a small fan installed in the opaque box containing the pit. We then find that the surface of the pit will be only 6-7 K warmer than the surrounding air, translating to just 2% difference in temperatures. With plutonium's heat conductivity at ~6 W/m/k, it can be shown that the pit will be at approximately uniform temperature.

Similarly, we can determine the average temperature of the WGPu encrypting filter as described in Supplementary Note 1. For a cylinder of length $z$, radius $R$, and a total heat production rate $P$ the steady state temperature is

$$T_0 - T_\infty \approx \frac{P}{h(2\pi Rz + 2\pi R^2)},$$

where $h = 20$ W/m$^2$/K is the heat transfer coefficient of air flowing at 1 m/s[4]. Given the cylinder's size, it is possible to show that the Biot number is $Bi \ll 1$, allowing for the above approximation. Here we would like to point out that the encrypting filter does not have to be monolithic. To address worries of criticality and to improve heat removal the 6 cm long cylinder can be split into four identical 1.5 cm long, 450 gram cylinders, aligned with each other and with the beam. For one of these cylinders the temperature difference with the ambient temperature is then $T_0 - T_\infty = 7$ K,

which is less than 1 K higher than the temperature of the pit itself.

The Doppler broadening function for the resonance lines has a Gaussian form, with a standard deviation of $\Delta = 2\sqrt{\frac{Ek_{\mathrm{B}}T}{A}}$, see Hébert, page 35[5]. The intrinsic resonances are typically $\mathcal{O}(0.2eV)$ wide, while $\Delta \sim 0.2$eV. To understand its dependence on small variations in temperature, we can write $\Delta \propto \sqrt{T} \approx T_0(1 + \frac{\delta T}{2T_0})$, where we have Taylor expanded the square root around $T_0 = 306.5$ K, the average temperature between the pit and the encrypting filter. We then conclude that the $\delta T = \pm 0.5$ K difference in temperatures of the pit and the filter will translate to thermal broadening differences of $\sim \pm 0.08$ %. It is then reasonable to assume that the difference in the total line widths between the pit and the encrypting filter is too small to be detectable in a measurement whose precision is fundamentally limited both by statistics and energy resolution.

However, if any doubts persist about differences larger than the one determined above, then the host can resolve this by simply mounting heating tapes to either one of the two objects, and perform temperature-controlled heating as a way of guaranteeing that the two objects are in fact at exactly the same temperature.

**Supplementary Note 3**

The concept of Zero Knowledge proof requires that the verifier learns nothing new about the object undergoing verification. In the context of an inspection exercise, it is important to show that the inspectors will learn nothing of value as a results of their observations. In our prior work[2], Section Isotopic Information Security, we showed that the inspectors will be unable to make any isotopic

inferences about the Treaty Accountable Item (TAI). The experiments performed in this study focused on a sensitivity analysis, with the goal to show that hoaxes can be detected, and thus did not involve additional measurements testing the physical cryptography of the test. However open beam data with no target or encrypting filters was collected. A Geant4[6] computational model of the experimental facility uses this data as an input, allowing to simulate the transmitted neutron signal for the weapon pit and encrypting filter geometries described in Supplementary Note 1. For simplicity, only the $^{239}$Pu and $^{240}$Pu isotopes were used. The Geant4 used the JEFF-3.2 neutron libraries[7]. Supplementary Figure 2 plots a spectrum of the open beam neutron data. The data shows that 821504 neutron counts were registered in the 5 minute long measurement. Supplementary Figure 3 shows a visualization of the Geant4 model. The $^{6}$Li glass detector used in this experiment was placed 14.573 meters from the photoneutron target, with the targets placed 110 cm upstream from the detector. The flight path was located in a 2 m wide concrete tunnel. The 6 cm encrypting filter is split into four 1.5 cm section, for reasons described in Supplementary Note 2. Since the data already encodes the detector's intrinsic energy-dependent efficiency the detector in the simulation is modeled at 100% efficiency. The experimental data is histogramed in Supplementary Figure 2.

The main arguments presented in Supplementary Note 1 are based on the assumption that the transmitted neutron signal is only sensitive to the total combined thickness or areal density of the pit and the encrypting filter. This implies that the total transmitted signal cannot be used to infer specifics about the pit or the filter alone. To prove this, we have ran a series of simulations where we vary the pit and filter dimensions and isotopics such that the combined areal density and the combined isotopic enrichment is kept constant. In all simulations 821504 neutron counts were

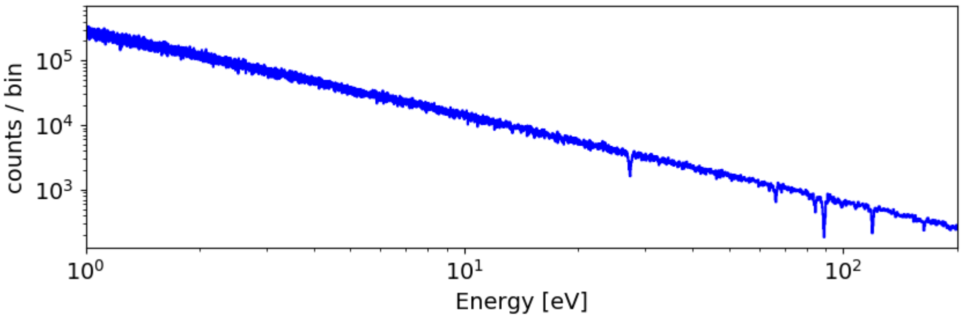


Supplementary Figure 2: Spectrum of open beam neutron data from the experiment. The observed absorption lines correspond to the resonances of the isotopes of a thin cadmium foil placed near the photoneutron target for filtering out the thermal neutrons.

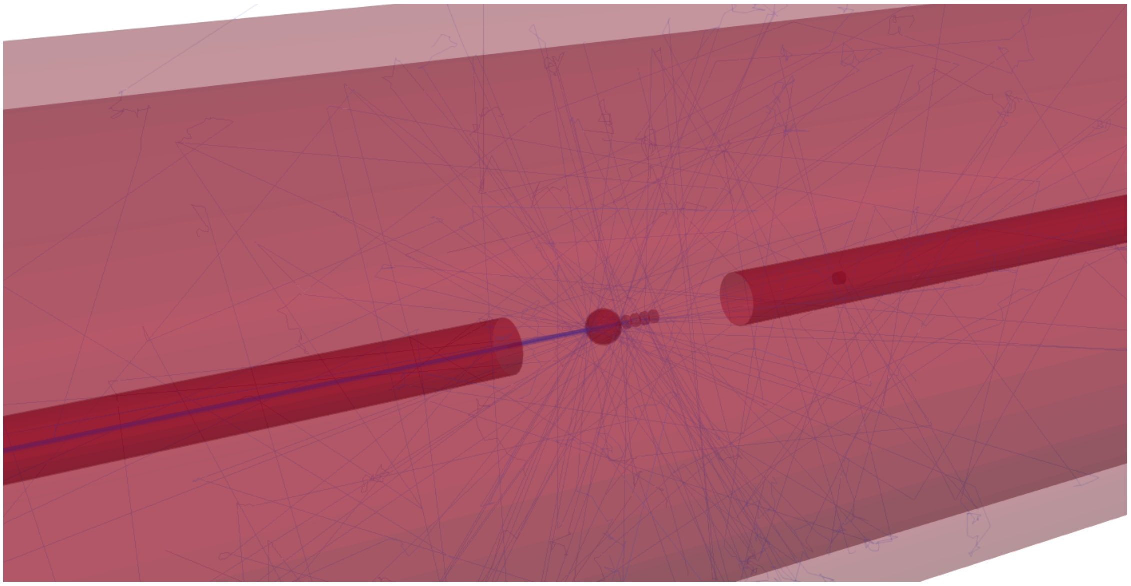

Supplementary Figure 3: Rendering of the Geant4 model. It shows the beam line, the WGPu pit, and the encrypting filter.

incident, sampled from the experimental data plotted in Supplementary Figure 2 and corresponding to a 5 minute measurement at the experimental facility used in this study. Supplementary Figure 4 shows the spectral results of the simulations in the 0-200 eV and 0-60 eV ranges. Supplementary Table 1 lists the various scenarios and the results of the output comparisons in a $\chi^2$ test. All of these have the commonality of combined areal density as traversed by the beam. The $\chi^2$ tests show that the results for the various combinations produce statistically identical spectral signals, indicating that the signal cannot be used to infer information about the pit alone.

The principle of mass encryption also applies to the isotopics of the pit. While the inspectors may be able to infer the combined, average isotopic enrichment of the pit and the encrypting filter, that average translates to a very large range of possible pit-specific enrichment values. To prove this, we performed simulations for a variety of enrichment levels for the pit and the filter. The results show that given a freedom of variation on the filter enrichment, the pit enrichment may vary in the range of 75-98%, yielding statistically identical result, as presented in Supplementary Table 2 and Supplementary Figure 5. If the signal is independent of the pit enrichment then the signal cannot be used to infer it. Hence as long as the inspector doesn't know the enrichment of the filter they cannot make inferences about the isotopic enrichment of the pit. At the same time the technique shows that changing the pit enrichment from 93% to the 60%, typical for reactor grade plutonium (RGPu), while keeping the encrypting filter the same, as would be the case in the actual verification exercise, would result in a statistically significant difference. Such a large observed difference would result in the rejection of the hoax. All simulation results reflect a 5 minute-long measurement at the experimental facility where the data of this study was acquired. At the

**a**

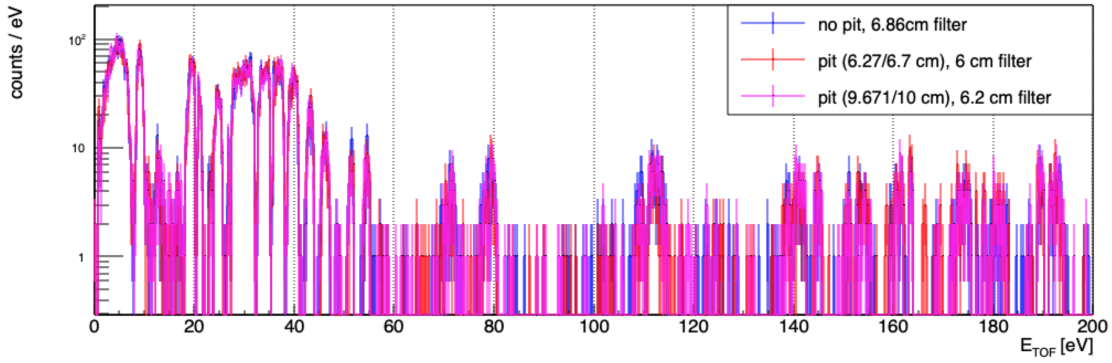


**b**

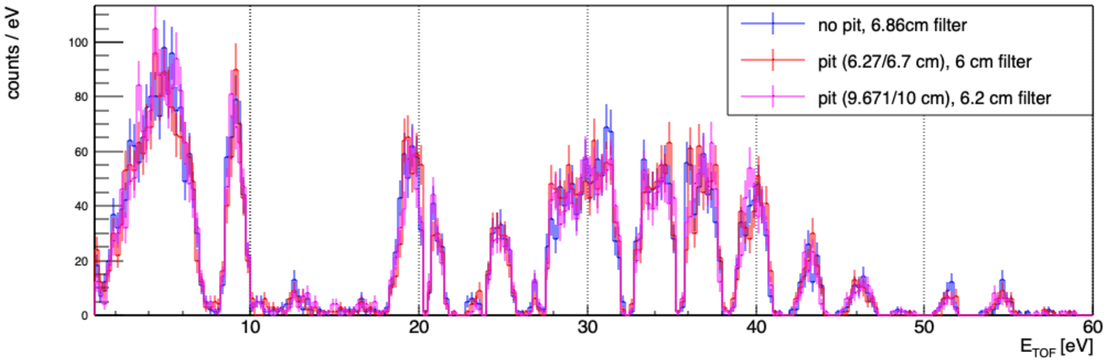


Supplementary Figure 4: Results of the simulations of various pit masses. The spectra for three scenarios are overlaid for 0-200 eV (**a**) and 0-60 eV (**a**) ranges, showing overall statistically identical distributions. The $\chi^2$ test results can be found in Supplementary Table 1.

same time, the simulations of hoaxing scenarios – one involving a shift in isotopics, and another involving a shift in geometry – are readily detectable, as can be seen in Supplementary Figure 6 and tabulated in Supplementary Table 2. This result shows that a 5 min long measurement is easily sensitive in isotopic changes of the WGPu/RGPu scale, and geometric changes of $\sim$2 mm. A sensitivity to smaller changes can be achieved by increasing the measurement times, the neutron beam flux (e.g. by increasing the beam current or by shortening the distance to the photoneutron source), by choosing a more efficient photoneutron source, or by selecting a larger detector. We can use the results from the above simulations to estimate the experimental conditions necessary for achieving diversions involving mass removals as small as 0.1 mm. The $\chi^2$ is defined as $\chi^2 = \sum(\Delta c_i/\sigma_i)^2$, where $\Delta c_i$ is the count difference in a particular bin in Supplementary Figure 6 and $\sigma_i$ is the uncertainty in that difference. Assuming that the difference changes linearly with the difference in thicknesses (true for small changes relative to the total thickness, i.e. 2$\rightarrow$0.1 mm relative to 66.8 mm), a 20$\times$ decrease in the difference of thicknesses will translate to 20$\times$ decrease in $\Delta c_i$, in average. This decrease in $\Delta c_i$ needs to be compensated by a similar decrease in $\sigma_i$ in order to maintain the same statistical significance. This then translates to a statistical volume which needs to be 400$\times$ larger. For the particular experimental facility used in this study, this would mean the following changes: bring the measurement point from the 15 m flight path distance to 5 meters, which will increase the neutron flux by 9$\times$; increase the electron beam current by 2$\times$; increase the measurement times from 5 min to approximately 2 hrs.

A possible source of false positives may be introduced by the $\sim$14.3 year half life $\beta$-decay of $^{241}$Pu into $^{241}$Am, causing differences between honest pits of different age. Since $^{241}$Pu constitutes

only $\sim$0.3% of WGPu [3],this is a small effect, in particular given that only 8.6 mm of the combined thickness of 68.6 mm is the actual pit's thickness. For this scenario, assuming a "fresh" filter and a pit of WGPu which is 10 years old, it can be shown that americium makes up only 1 part per 6650 of the areal density. To verify that the scale of this effect is insignificant we performed additional simulations, where we compared a "fresh" pit with no americium, to the one that has been "aged" for 10 years and has had some buildup of americium. Supplementary Figure 7 plots the two transmission spectra. These spectra are slightly different from the results of other simulations. This is only due to a more precise isotopic modeling, based on Table 1 of Mark et al.[3]. The spectral comparison shows an overall agreement, and indicates that the differences of americium content should not be a cause of false positives for pits with age differences of 10 years or less. Furthermore, the statistically identical spectra indicate that there is no americium-dependent information content in the spectra themselves.

The analysis above is relatively simple, and while some sophisticated inference analyses are theoretically possible, any concerns about information security - as well as the necessary fixes - related to americium and other impurities should be the focus of future research, and are outside of the scope of this study. For example, the use of a chopper, as described in the main body could be used to physically limit the spectrum to very specific ranges which do not include the americium lines. Also, the hosts can use filters with high content of americium, thus statistically diluting the americium-specific differences between genuine pits. Finally, the hosts may simply choose to use this technique to dismantle weapons of similar age as the golden copy. All these considerations and trade studies are part of the future research, and should be done with the classified knowledge

of the weapons age, structure, and composition.

**Supplementary Note 4**

The method described in this study uses nuclear resonance phenomena as a basis for physical cryptography. The main strength of resonance phenomena is that it involves signals that are unique to individual isotopes, allowing to achieve a one to one map from the measurement space to isotope space. However in order for this map to be truly one to one, the resonance energies for uranium and plutonium need to be unique to their isotopes. While this study uses the broad energy range of [0,200] eV as part of the proof of concept demonstration, the plutonium and uranium isotopes' strongest and most clearly separated transitions are located in the narrower [0,10] eV range. This is also the range where TOF techniques produce the highest resolution.

We thus focus on [0,10] eV range for a test of uniqueness. This means searching for possible duplication of uranium and plutonium resonances in other, commonly available elements. Supplementary Table 3 lists the major isotopes of the elements which have resonances in the above mentioned range. The data was acquired from the National Nuclear Data Center(NNDC)[8], and is sorted by resonance energy.

An inspection of the table reveals that there are many isotopes that have resonances in this range. Furthermore, some of these isotopes have resonances that are close in energy to some plutonium or uranium resonances. However in order to achieve an isotopic hoax, it is not sufficient to replicate an individual line. The elements or the combination of elements in a potential hoaxing

**a**

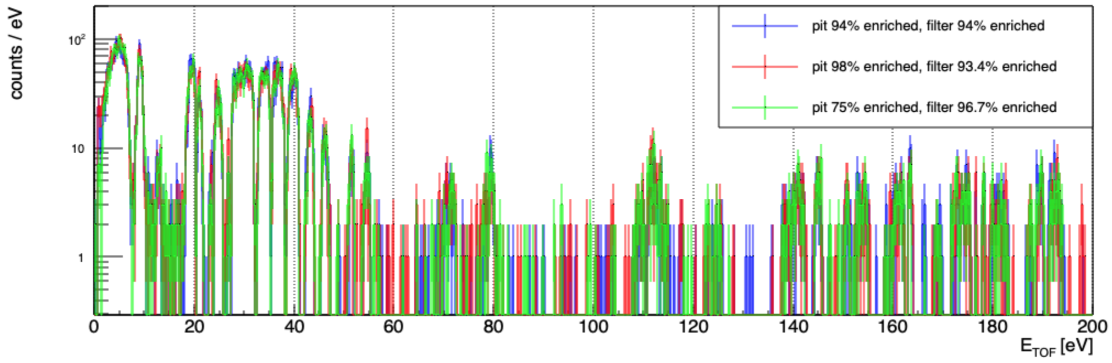


**b**

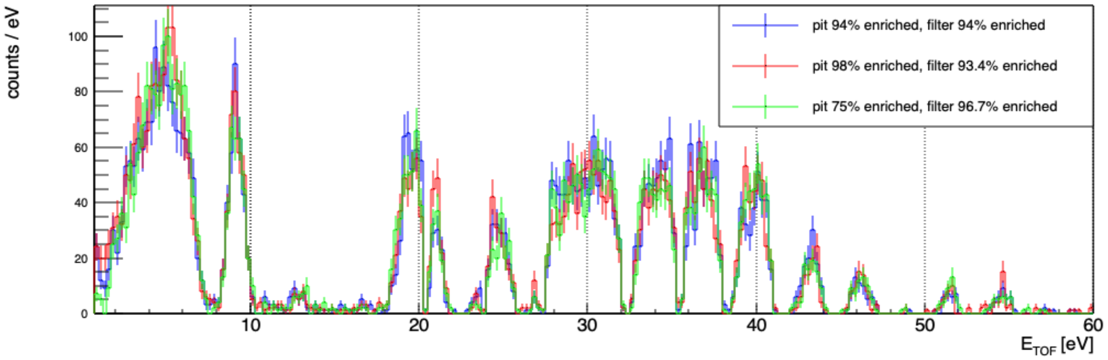


Supplementary Figure 5: Results of the simulation for various isotopic enrichments. The spectra for the three scenarios are overlaid for 0-200 eV (**a**) and 0-60 eV (**b**) ranges, showing overall statistically identical distributions. The $\chi^2$ test results can be found in Supplementary Table 2.

**a**

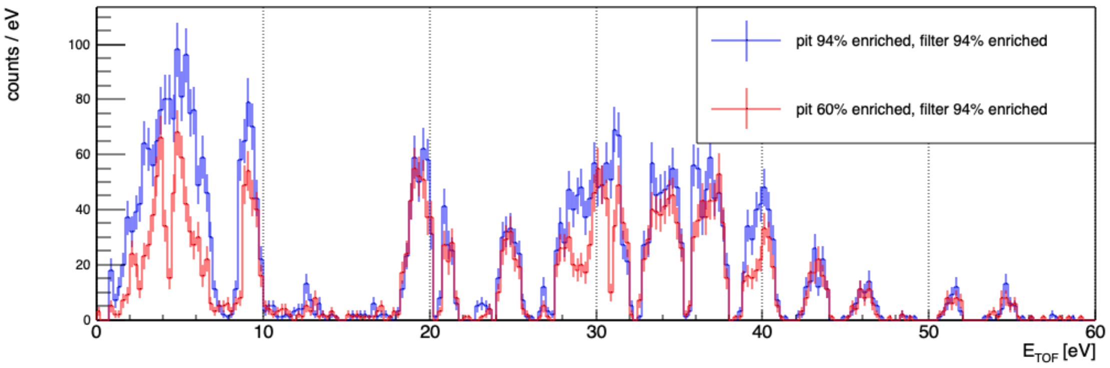


**b**

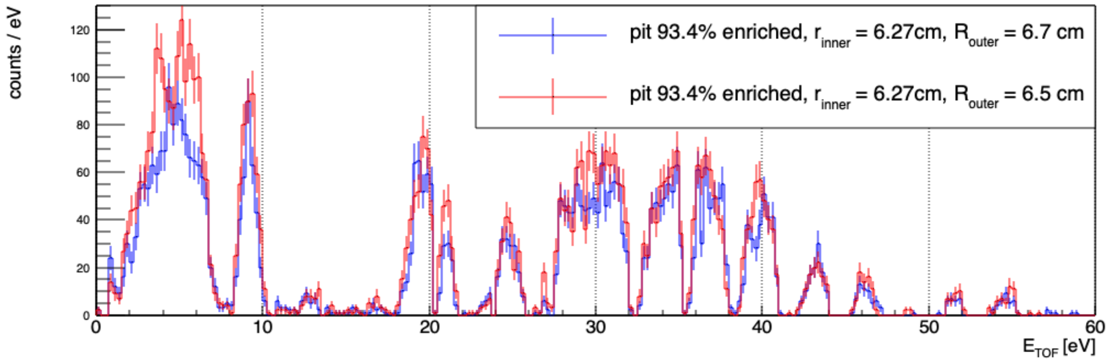


Supplementary Figure 6: Results of the simulation for two hoaxing scenarios – isotopic replacement, and geometric change. A replacement of the WGPu pit with a RGPu while holding the filter at WGPu enrichment readily reveals the hoax (**a**). Technique can also detect hoaxes involving correct isotopics but of a 2 mm smaller size (**b**). The $\chi^2$ test results can be found in Supplementary Table 2.

| description | radii, inner/ outer [cm] | $d_{\text{filter}}$ [cm] | total [cm] | pit [kg] | 0-200 eV $\chi^2$ | NDF | $p$ | 0-60 eV $\chi^2$ | NDF | $p$ |
|---|---|---|---|---|---|---|---|---|---|---|
| no pit | 0 / 0 | 6.86 | 6.86 | 0 | - | - | - | - | - | - |
| Black Sea model | 6.27/6.7 | 6 | 6.86 | 3.4 | 263 | 344 | 1.0 | 157 | 170 | 0.75 |
| 6kg pit | 9.671/10 | 6.202 | 6.86 | 6 | 254 | 338 | 0.99 | 159 | 172 | 0.75 |

Supplementary Table 1: The various pit and encrypting filter geometries simulated. Statistical comparisons are performed relative to the scenario in the first row. The statistical $\chi^2$ tests were performed for the 0-60 eV and 0-200eV energy ranges, with the probabilities ($p$ values) indicating a statistically identical signals.

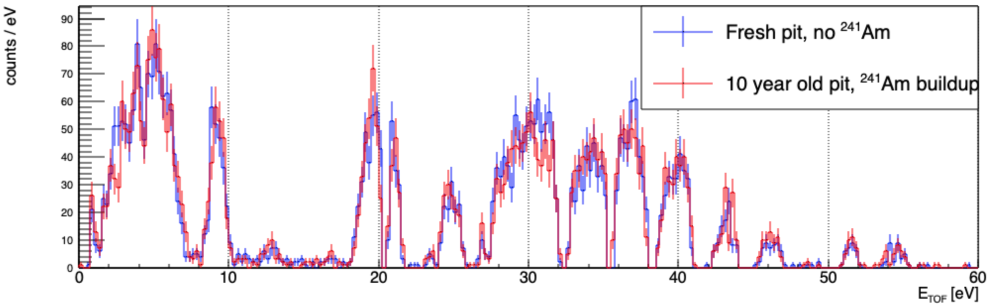


Supplementary Figure 7: Comparisons of a fresh WGPu pit with no $^{241}$Am to a ten year old pit. In this simulation the isotopic ratios were taken fro Table 1 of Mark et al.[3]. The overall comparison yields a $\chi^2/NDF = 161/170$, with $p = 0.68$, indicating no false positive due to the differences in americium content.

| | | enrichment | | 0-200 eV | | | 0-60 eV | | |
|---|---|---|---|---|---|---|---|---|---|
| description | pit | filter | combined | $\chi^2$ | NDF | $p$ | $\chi^2$ | NDF | $p$ |
| WGPu | 94% | 94% | 94% | - | - | - | - | - | - |
| super grade | 98% | 93.4% | 94% | 274 | 342 | 0.99 | 173 | 169 | 0.40 |
| low grade | 75% | 96.7% | 94% | 253 | 330 | 1.0 | 158 | 165 | 0.64 |
| **RGPu hoax** | 60% | 94% | **89.7%** | 698 | 342 | $\mathbf{\sim 10^{-26}}$ | 573 | 169 | $\mathbf{\sim 10^{-45}}$ |
| **Size hoax** | 94% | 94% | 94% | 399 | 340 | **0.015** | 291 | 171 | $\mathbf{\sim 10^{-8}}$ |

Supplementary Table 2: The various pit and encrypting filter enrichments simulated. Statistical comparisons are performed to the scenario in the first row. The results of the simulation indicate that as long as the combined enrichment is constant, there is no statistically significant difference in the signal. At the same time a RGPu hoax is readily exposed. The size hoax, which involves a pit of the correct enrichment but of an outer radius 2 mm smaller than the reference object, is also detected.

| Element | Major Isotope | E [eV] | GN | GG | GFA | GFB |
|---|---|---|---|---|---|---|
| iridium | 193Ir | 1.298000+0 | 8.723040-2 | 7.304000-4 | 8.650000-2 | 0.000000+0 |
| indium | 115In | 1.457000+0 | 7.498182-2 | 2.981818-3 | 7.200000-2 | 0.000000+0 |
| **plutonium** | **241Pu** | **1.725295+0** | **2.074000-6** | **4.030000-2** | **6.786000-2** | **2.823000-1** |
| rhenium | 185Re | 2.156000+0 | 5.772900-2 | 2.828600-3 | 5.490000-2 | 0.000000+0 |
| **uranium** | **235U** | **3.615605+0** | **4.213500-5** | **3.766200-2** | **-5.476900-2** | **-2.034000-5** |
| indium | 115In | 3.850000+0 | 8.137666-2 | 3.766667-4 | 8.100000-2 | 0.000000+0 |
| tungsten | 182W | 4.150000+0 | 1.550000-3 | 4.800000-2 | 0.000000+0 | 0.000000+0 |
| tantalum | 181Ta | 4.280000+0 | 5.690000-2 | 3.900000-3 | 5.300000-2 | 0.000000+0 |
| **plutonium** | **241Pu** | **4.285520+0** | **5.758000-4** | **3.471000-2** | **2.780000-2** | **0.000000+0** |
| rhenium | 187Re | 4.416000+0 | 5.491800-2 | 3.180000-4 | 5.460000-2 | 0.000000+0 |
| **plutonium** | **241Pu** | **4.587276+0** | **4.787000-4** | **3.677000-2** | **-1.922000-2** | **1.211000-1** |
| **uranium** | **235U** | **4.849738+0** | **5.245800-5** | **3.813800-2** | **-1.539900-4** | **-4.880600-3** |
| gold | 197Au | 4.890000+0 | 1.520000-2 | 1.240000-1 | 0.000000+0 | 0.000000+0 |
| silver | 109Ag | 5.190000+0 | 1.427333-1 | 1.273333-2 | 1.300000-1 | 0.000000+0 |
| iridium | 191Ir | 5.360000+0 | 8.744000-2 | 5.440000-3 | 8.200000-2 | 0.000000+0 |
| **plutonium** | **241Pu** | **5.813320+0** | **2.774000-3** | **6.007000-2** | **-1.180000+0** | **1.805000-1** |
| cesium | 133Cs | 5.910000+0 | 1.264100-1 | 6.410000-3 | 1.200000-1 | 0.000000+0 |
| rhenium | 185Re | 5.920000+0 | 6.926400-2 | 2.640000-4 | 6.900000-2 | 0.000000+0 |
| iridium | 191Ir | 6.119000+0 | 8.370666-2 | 7.066667-4 | 8.300000-2 | 0.000000+0 |
| **uranium** | **235U** | **6.388841+0** | **2.419300-4** | **4.268300-2** | **4.491100-4** | **1.181300-2** |
| **uranium** | **238U** | **6.673491+0** | **1.475792-3** | **2.300000-2** | **0.000000+0** | **9.990000-9** |
| **plutonium** | **241Pu** | **6.945672+0** | **6.201000-4** | **3.546000-2** | **-1.075000-1** | **4.514000-4** |
| **uranium** | **235U** | **7.079407+0** | **1.122900-4** | **3.904800-2** | **1.812000-2** | **-1.473300-2** |
| rhenium | 185Re | 7.220000+0 | 5.619100-2 | 1.191400-3 | 5.500000-2 | 0.000000+0 |
| tungsten | 183W | 7.640000+0 | 1.660000-3 | 8.299990-2 | 0.000000+0 | 0.000000+0 |
| hafnium | 178Hf | 7.786500+0 | 1.068300-1 | 5.383000-2 | 5.300000-2 | 0.000000+0 |
| **plutonium** | **239Pu** | **7.815800+0** | **7.920000-4** | **3.775000-2** | **-4.475000-2** | **0.000000+0** |
| **plutonium** | **241Pu** | **8.622176+0** | **7.797000-4** | **3.358000-2** | **8.195000-4** | **5.920000-2** |
| **uranium** | **235U** | **8.760378+0** | **9.661400-4** | **3.769200-2** | **1.010300-1** | **-6.253200-3** |
| iridium | 193Ir | 9.068000+0 | 9.224001-2 | 2.240000-3 | 9.000000-2 | 0.000000+0 |
| iridium | 191Ir | 9.068000+0 | 8.312000-2 | 3.120000-3 | 8.000001-2 | 0.000000+0 |
| indium | 115In | 9.070000+0 | 8.153636-2 | 1.536364-3 | 8.000000-2 | 0.000000+0 |
| **uranium** | **235U** | **9.270811+0** | **1.195900-4** | **4.135300-2** | **2.224300-2** | **5.140100-2** |
| **uranium** | **235U** | **9.649351+0** | **5.924000-4** | **3.908000-2** | **2.037000-1** | **1.707000-3** |
| iridium | 191Ir | 9.894000+0 | 9.200000-2 | 1.000000-3 | 9.100000-2 | 0.000000+0 |
| **uranium** | **235U** | **9.938550+0** | **1.894000-3** | **5.661000-2** | **8.886000-1** | **2.920000-4** |
| **plutonium** | **239Pu** | **1.092800+1** | **1.795000-3** | **3.612000-2** | **-1.540000-1** | **0.000000+0** |

Supplementary Table 3: A listing of elements and isotopes with resonances sorted by energy. The energy range is $1$ eV $\leq E \leq 11$ eV. The relevant isotopes are boldfaced for comparison. Data has been acquired from and is in the format of NNDC[8]. GN and GG refer to the neutron scattering and capture widths, in eV. GFA and GFB refer to the fission widths, in eV, as defined by Reich-Moore multilevel resonance formalism.

mix need to have a combination of lines that simultaneously reproduce plutonium's resonances' energies, widths, and relative strengths. At the same time this combination of elements should not give rise to any additional lines. For example, to emulate $^{241}$Pu and $^{239}$Pu lines at 1.73, 4.3, 4.6, 5.8, 6.9, 7.8, 8.6, and 10.1 eV the host could try to use the 1.46, 4.3, 4.4, 5.91, 7.2, 7.8, 9.06, and 9.9 eV lines of indium, tantalum, rhenium, cesium, and hafnium, respectively. However, this mix of elements will also produce lines at 1.3, 3.8, 5.36, and 6.1 eV - lines that are missing in plutonium isotopes. Furthermore, at higher energies these elements manifest additional lines not seen in plutonium or uranium. These non-fissile lines in the hoax will induce significant differences in measurements between the hoax described above and a plutonium pit, resulting in a rejection of the hoax.

While this analysis indicates the difficulty of achieving isotopic hoaxing by combination of other elements, future research needs to focus on the problem of uniqueness, i.e. systematically proving that no combination of materials can simultaneously reproduce the combined cross section shape of plutonium's isotopes. Since future embodiment of this technique may involve more compact experimental setups with an energy resolution that is lower than the one observed in this study, the analysis of uniqueness also needs to take into account energy resolution for a particular experimental setting.

Note that Supplementary Table 3 only includes elements that are easily available and are not difficult to handle. The table does not list the resonances of $^{241}$Am, for example, as americium is too radioactive to be used in any hoaxing manipulation that requires $\sim$kg quantities of materials.

## Supplementary References


1. Fetter, S., Cochran, T. B., Grodzins, L., Lynch, H. L. & Zucker, M. S. Gamma-ray measurements of a Soviet cruise-missile warhead. *Science* **248**, 828–834 (1990).

2. Hecla, J. J. & Danagoulian, A. Nuclear disarmament verification via resonant phenomena. *Nature communications* **9**, 1259 (2018).

3. Mark, J. C., von Hippel, F. N. & Lyman, E. Explosive properties of reactor-grade plutonium. *Science & Global Security* **17**, 170–185 (2009).

4. Bergman, T. L., Incropera, F. P., DeWitt, D. P. & Lavine, A. S. *Fundamentals of heat and mass transfer* (John Wiley & Sons, 2011).

5. Hébert, A. *Applied reactor physics* (Presses inter Polytechnique, 2009).

6. Agostinelli, S. *et al.* GEANT4: A simulation toolkit. *Nucl. Instrum. Meth.* **A506**, 250–303 (2003).

7. Mendoza, E., Cano-Ott, D., Koi, T. & Guerrero, C. New standard evaluated neutron cross section libraries for the GEANT4 code and first verification. *IEEE Transactions on Nuclear Science* **61**, 2357–2364 (2014).

8. Evaluated Nuclear Data File Retrieval and Plotting. http://www.nndc.bnl.gov/sigma/. Accessed: 2019-06-23.
